# Real-Time Reverse Transcription Multienzyme Isothermal Rapid Amplification for Rapid Detection of African Horse Sickness Virus

**DOI:** 10.1155/tbed/1852368

**Published:** 2025-01-13

**Authors:** Chaohua Huang, Jianchang Wang, Zhouxi Ruan, Jiang Wu, Yanxing Lin, Chenfu Cao, Junxing Yang, Qiaoyu Weng, Ye Jin, Peng Chen, Qunyi Hua

**Affiliations:** ^1^Laboratory of Animal Quarantine, Animal and Plant Inspection and Quarantine Technology Center of Shenzhen Customs, Shenzhen 518045, Guangdong Province, China; ^2^Laboratory of Animal Quarantine, Technology Center of Shijiazhuang Customs, Shijiazhuang 050051, Hebei Province, China; ^3^Department of Product Development, Shenzhen Biolove Technology Co., Ltd, Shenzhen 518120, China; ^4^Department of Product Development, Hu Nan Project Bioscience Ltd, Changsha 410013, China

**Keywords:** african horse sickness virus, rapid detection, real-time RT-MIRA, screening

## Abstract

African horse sickness (AHS) is an acute infectious disease of equids caused by the AHS virus (AHSV), which can cause up to 90% mortality in naive horses. Reliable and rapid diagnosis is crucial for the surveillance and control of AHSV. As one of the AHSV detection methods recommended by World Organization for Animal Health (WOAH), the RT-qPCR assay has the drawbacks such as complex operation, expensive instruments, and long detecting time, which limit its application in simple laboratories or outdoors. In this study, a real-time reverse transcription multienzyme isothermal rapid amplification (RT-MIRA) assay was established to detect AHSV. Primers and exo-probes were designed, synthesized, and screened based on the conserved regions of the AHSV *Seg-7* gene. A series of experiments were conducted to evaluate the performances of the established real-time RT-MIRA for detecting AHSV. The valid testing results showed that this method was highly specific for the detection of AHSV, without exhibiting any cross-reactivity towards other equine viruses or other Orbivirus; its limit of detection (LOD) was 10 copies/μL, which was consistent with that of RT-qPCR, meaning it had good sensitivity for detecting AHSV. Furthermore, the real-time RT-MIRA for AHSV performed good repeatability, and its standard curve exhibited good linearity with a correlation coefficient of *R*^2^ = 0.9898, which indicated that the established method could be used for the quantitative detection of ASHV. As no AHS infection cases have been reported in China, 120 simulated clinical samples were tested by the real-time RT-MIRA and RT-qPCR for AHSV, which results showed there was a significant correlation between the two assays, with a *κ* value of 0.966 and an *R*^2^ value of 0.9576. Parallel detection of 396 equine blood samples and 1760 Culicoides by this method and the RT-qPCR showed that all samples were negative for AHSV. Furthermore, the results of the real-time RT-MIRA could be judged by naked eyes under a portable equipment with blue light (480 nm). In conclusion, the real-time RT-MIRA for AHSV was specific and sensitive and had the advantages of convenient operation, visualization, no need for special equipment, and could be a reliable tool for rapid screening and detection of AHSV in field or border ports.

## 1. Introduction

African horse sickness (AHS) is a noncontagious vector-borne disease caused by the AHS virus (AHSV) [1], which is transmitted between hosts by hematophagous midges of the genus *Culicoides* [2]. Susceptible hosts to AHSV are horses and other equids (mules, donkeys, and zebras) [3]. AHS is highly lethal in naïve horses with high mortality rates (>90%) [4]. Donkeys are more resistant to the disease, with less severe clinical signs and death expected in around 5%–10% of cases [5]. In general, zebras are resistant to AHSV, exhibit asymptomatic infection, therefore, are considered to be the hosts maintaining for this disease [6]. AHS is generally endemic in countries of the southern Africa and has been recorded previously in Morocco, Spain, Cyprus, Portugal, India, Iran, and Pakistan [7–9]. However, global warming has expanded the range and duration of AHSV vectors activities, which is likely to expand the range of the AHS epidemic [7]. Recent outbreaks of ASH in Thailand and Malaysia in 2020 indicate that AHS may have escaped geographical limitations and entered Southeast Asia [10].

AHSV is a member of the genus *Orbivirus* in the family Reoviridae [11], which genome consists of 10 double-stranded RNA segments (*Seg-1*–*Seg-10*), encoding seven structural proteins (VP1–VP7) and four nonstructural proteins (NS1, NS2, NS3/NS3A, and NS4) [12, 13]. AHSV has a similar morphological structure to the bluetongue virus (BTV). Its outer capsid layer consists of two major structural proteins VP5 and VP2 [14], while the core particle of ASHV are composed of VP1, VP3,VP4, VP6, and VP7 [8, 15]. As the primary determinant of the AHSV serotype, VP2 is the most variable antigen and the main target of virus-neutralizing antibodies [16, 17]. To date, there are nine different serotypes of AHSV (AHSV-1–AHSV-9) identified based on VP2, and the virulence of different types is different [18, 19]. VP3 and VP7, encoded by Seg-3 and Seg-7, are highly conserved group-specific antigenic proteins in AHSV [20]. Furthermore, VP7 is the main target of enzyme-linked immunosorbent assay (ELISA) assay and molecular biology assay for AHSV [21–23].

Due to its associated high mortality to naïve horse populations and the potential for rapid international spread through *Culicoides* biting, a sensitive and reliable assay for rapid detection AHS is urgently required to prevent, control, and eliminate this disease. Laboratory diagnostics for AHS include etiological and serological tests. Most of the serological methods detecting AHS are complement fixation (CF), virus neutralization (VN), and ELISA methods, including blocking ELISA, indirect ELISA, and competitive ELISA. Several laboratory etiological methods have been applied to detect AHSV, including virus isolation (VI), immunofluorescence, antigen-detecting ELISA, and molecular biology techniques. Although the VI is the classic gold-standard method, its operation procedure is complex and takes about 2–5 days, not suitable for rapid diagnosis. Relatively, molecular biological detection techniques such as RT-PCR and real-time RT-PCR are more widely used to detect ASHV. Among them, an agarose gel–based RT-PCR developed by Zientara et al. [24] and the real-time RT-PCR methods established by Aguero et al. [25] and Guthrie et al. [26], respectively, are recommended by the World Organization for Animal Health (WOAH). The conventional RT-PCR or real-time RT-qPCR for determination of AHSV type is mostly based on the detection of *Seg-2*, occasionally aimed at the specific regions of *Seg-1* or *Seg-3*. For rapid DNA/RNA detection, several techniques based on isothermal amplification were developed, such as loop-mediated isothermal amplification (LAMP) [27], rolling circle amplification (RCA) [28], and recombinase polymerase amplification (RPA) [29]. The rapid AHSV RT-LAMP assay based on AHSV *Seg-7* has sufficiently high specificity and sensitivity for the detection of AHSV [30].

As a novel isothermal amplification technology, the multienzyme isothermal rapid amplification (MIRA) assay utilizes the synergistic role of multiple enzymes or functional proteins (DNA polymerase, recombinase, helicase, and single-stranded binding protein) to achieve rapid amplification at an isothermal condition. The difference of MIRA with RPA is that it uses a recombinase derived from Streptomyces azure, which improves amplification efficiency and stability and reduces interference [31]. The advantages of reverse transcription (RT)-MIRA/MIRA are rapid reaction and low reaction temperature, which only takes 20–30 min to complete the reaction at 33–42°C, while the RT-LAMP/LAMP reaction needs more than 1 h at 60–65°C. In the meantime, there are several patterns to analyze the RT-MIRA/MIRA amplification products, such as agarose gel or capillary electrophoresis, fluorescence detection, and lateral flow strip. So far, the RT-MIRA/MIRA technique has been used in detection of human, animal, and plant pathogens, such as SARS-CoV-2, hepatitis a virus, classical swine fever virus, pseudorabies virus (PRV), sweet potato feathery mottle virus, and sweet potato chlorotic stuntvirus [32–36]. However, as yet there has not been an RT-MIRA method developed for the detection of AHSV.

In the present study, a real-time RT-MIAR assay based on a highly conserved region within AHSV *Seg*-7 was established. As a sensitive and reliable method for rapid detection of AHSV, the results of this method in simulated samples, field samples and *Culicoides* samples were highly consistent with the results of RT-qPCR recommended by WOAH.

## 2. Material and Methods

### 2.1. Plasmid and Virus Nucleic Acid

The plasmid pMD18-AHSV-vp7 containing AHSV *Seg-7* gene (GenBank: KT030596.1), BTV-1 RNA, epizootic hemorrhagic disease virus (EHDV) RNA, and PRV RNA was preserved at our lab. Equine infectious anemia virus (EIAV) RNA, equine viral arthritis virus (EVAV) RNA, and equine influenza virus (EIV) RNA were purchased from Harbin Veterinary Research Institute, Chinese Academy of Agricultural Sciences.

### 2.2. Designing, Synthesizing, and Screening of Primers for RT-MIRA

For the MIRA/RT-MIRA, the primer length is usually 30–35 bp and the optimal length of the amplicon is 150–500 bp. Five specific primer pairs of RT-MIRA amplification were designed based on the conserved region of the *Seg*-7 gene (GenBank: KT030596.1) of AHSV (Table 1) by the design software (PrimerPrimer, Version 5.0) and synthesized by Sangon (Shanghai, China). For screening the best RT-MIRA amplification primer pairs, amplification reactions were conducted using the RT-MIRA kits (AMP-Future Biotech, Cat#: WLRB8207KIT) which provide Buffer A (PEG 3wt%, Tris 30 mM), Buffer B (Mg(Ac)_2_ 280 mM) and reaction tubes containing freeze-dried powder consisting of DNA polymerase, recombinase, helicase, single-stranded binding protein, reverse transcriptase, polyA, and dNTP. A RT-MIRA reaction mixture of 29.4 μL Buffer A, 2 μL of each F/R primers (10 μM), 13.1 μL of ddH_2_O, and 1 μL of the plasmid pMD18-AHSV-vp7 was added to each reaction tube containing freeze-dried powder. After adding 2.5 μL of Buffer B into the reaction mix, the tube was closed, vortexed, centrifuged, and then placed in a thermostatic metal bath at 42°C for 30 min. The results of the amplification reaction were analyzed by a capillary electrophoresis device (Qiagen, QIAxcel Advanced System) to determine the best specific primer pairs for RT-MIRA amplification.

### 2.3. Exo-Probe Designing and Screening for Real-Time RT-MIRA

There are four sites of modification on the exo-probe: A dSpacer (tetrahydrofuran, THF) is labeled in the middle position approximately 35 nt from the 5′ end of the exo-probe as the recognition site for exonuclease; the upstream and downstream of the THF site is labeled with a fluorescent group and a quenched group, and a distance of 2–4 nt between the two groups; the 3′ end of the exo-probe is labeled with a modifying group, such as an amino group, phosphate group, or C3 Spacer [31]. Two AHSV-specific exo-probes based on the target regions of the best primer pairs in previous screening were synthesized by Sangon (Shanghai, China) (Table 1).

For screening the optimal group of primers and exo-probe, the real-time RT-MIRA kits (AMP-Future Biotech, Cat#: WLRE8208KIT) were used according to the manufacturer's instructions with some modifications [35], in which come with Buffer A (PEG 3 wt%, Tris 30 mM), Buffer B (Mg(Ac)_2_ 200 mM), and reaction tubes containing freeze-dried powder consisting of DNA polymerase, recombinase, helicase, single-stranded binding protein, reverse transcriptase, exonuclease, polyA, and dNTP. All subsequent real-time RT-MIRA experiments in this study were conducted using the real-time RT-MIRA kits from Amp-Future Biotechnology Co., Ltd. Each reaction tube with freeze-dried powder was added with the 50 μL reaction mixture of real-time RT-MIRA consisting of 29.4 μL Buffer A, 2.0 μL F/R primer (final concentration 400 nM), 2.5 μL Buffer B, 0.6 μL exo-probe (final concentration 120 nM), 1.0 μL nucleic acid template, and made up to 50 μL with nuclease-free H_2_O. Buffer B was added in the last step, and the reaction tubes were quickly turned upside down 5–6 times and rapidly centrifuged, then immediately incubated at 42°C for 20 min in ABI QuantStudio 5 qPCR system (ThermoFisher, USA). The fluorescence signals were collected every 30 s with the FAM channel.

### 2.4. Standard RNA Preparation for AHSV Dection Using Real-Time RT-MIRA and RT-qPCR

To provide a standard AHSV positive RNA template for subsequent assays, a pair of PCR primers was designed (Table 1), named RNA template-F and RNA template-R, whose upstream primer 5′ end contained a T7 promoter. Its amplification range was between the 641–1167 bp of the AHSV *Seg-7 ge*ne, which contain the target region of the optimized RT-MIRA primers and the detection region of the RT-qPCR assay for AHSV recommended by WOAH. Plasmid pMD18-AHSV-VP7 containing AHSV Seg-7 gene was used as the template for PCR amplification using the primer pair mentioned above, and then the double-stranded DNA obtained by PCR then served as the DNA template for in vitro transcription reactions with a HiScribe T7 High Yield RNA Synthesis Kit (NEB, Cat#: E2040S,). The product of RNA fragments was purified using a miRNeasy Mini Kit (50; Qiagen, Cat#: 217004). The purified RNA was measured by a spectrophotometer (NanoDrop 2000, ThermoFisher, USA), and diluted to 1 × 10^7^ copies/μL and stored at −80°C.

### 2.5. Optimization of Temperature in Real-Time RT-MIRA Reaction

To determine the optimal temperature of the real-time MIRA, the standard AHSV RNA was detected using the real-time RT-MIRA kits with the screened optimal primer/probe combination. The reaction temperature of the real-time RT-MIRA was set at 33, 35, 37, 39, and 42°C, respectively. The reaction time was 20 min, and the fluorescence of the FAM channel was collected every 30 s. The optimal temperature of the real-time MIRA was obtained by analysis of the amplification curves of different temperatures.

### 2.6. Specificity of the Real-Time RT-MIRA Assay

To evaluate the specificity of real-time RT-MIRA assay in the detection of AHSV, the RNA of BTV, EHDV, and other important equine viruses, including PRV, EIAV, EAV, and EIV, were tested by the developed real-time RT-MIRA of AHSV. The positive control was the standard RNA template mentioned above, and the negative control was ddH_2_O. The reactions were at 35°C for 20 min, and the fluorescence signals were collected every 30 s. Furthermore, a B-BOX Blue light LED epi-illuminator (Smobio, China) was used to detect the real-time RT-MIRA amplification products.

### 2.7. Sensitivity of the Real-Time RT-MIRA Assay

To determine the sensitivity of the real-time RT-MIRA assay for AHSV, the standard AHSV RNA template was diluted to 1 × 10^7^ copies/μL ~ 1 × 10^0^ copies/μL to detect by this method under optimized conditions. At the meantime, the WOAH-recommended RT-qPCR for AHSV was used to detect these templates in parallel, which were developed by Guthrie et al. [26] with some modifications. The RT-qPCR assay was conducted using the AgPath-ID One-Step RT-PCR Reagents (Applied Biosystems, Cat#: 4387424) in a 25-μL reaction, containing 12.5 μL of 2 × RTPCR buffer, 1.0 μL of 25 × RTPCR enzyme mix, 0.5 μL of each AHSV-RT-qPCR-F/AHSV-RT-qPCR-R primer (final concentration 200 nM), 0.3 μL of AHSV-RT-qPCR—probe (final concentration 200 nM), 1.0 μL of nucleic acid template, and 9.2 μL of nuclease-free H_2_O. The RT-qPCR was performed on an ABI QuantStudio 5 qPCR System (Applied Biosystems, USA) with the following conditions: 48°C (10 min), 95°C (10 min), and then 40 cycles of 95°C (15 s) and 60°C (45 s), and the flourescence was collected at 60°C. Each concentration of the serial-diluted AHSV RNA templates was tested five times by RT-qPCR and RT-MIRA, respectively. The standard curves of RT-qPCR and RT-MIRA were constructed based on the results (cycle threshold (Ct) values) of the two methods to compare their accuracy.

### 2.8. Compliance Testing of Simulated Samples

While ASH has never occurred in China, our lab did not have AHSV-positive samples. In order to evaluate the compliance of the established real-time RT-MIRA with RT-qPCR assay for AHSV, 120 simulated samples were prepared by randomly mixing the AHSV standard RNA with AHSV-negative horse or donkey blood samples. These simulated samples were tested in parallel with these two assays. The positive result was determined by the amplification curve and Ct value of the real-time RT-MIRA and RT-qPCR. The efficacy of these two assays were analyzed based on sensitivity, specificity, positive predictive value (PPV), and negative predictive value (NPV) as follows: sensitivity = TP/(TP + FN) × 100%; specificity = TN/(TN + FP) × 100%; PPV = TP/(TP + FP); and NPV = TN/(TN + FN) (TP, true positive; FP, false positive; TN, true negative; FN, false negative). The degree of agreement (*κ* value) between these two methods was calculated using SPSS software (version 26.0). In addition, the amplification products of the real-time RT-MIRA were observed under 480 nm blue light.

### 2.9. Testing for Field Samples and *Culicoides* Samples

To further evaluate the performance of this method, 256 horse blood samples and 140 donkey blood samples were collected from Yunnan province, Guangdong province, and Hebei province in China. The total RNA of samples was extracted by a MagMAX-96 Viral RNA Isolation kit (ThermoFisher, Cat#: AM1836) following the manufacturer's directions and a KingFisher Flex Purification Instrument (96 Deep Well Processor; ThermoFisher, USA) and tested in parallel with the established real-time RT-MIRA method and the RT-qPCR for AHSV.

From June to September 2023, *Culicoides* were collected by UV light traps in Shenzhen of Guangdong province, China, while the collection locations were set inside Guanming Farm, Safari Park, Xianhu Botanical Garden, and Mangrove Coastal Ecological Park of Shenzhen City. As described previously with some modifications [37], six battery-powered UV light traps (LTS-M02; Wuhan Lucky Star Medical Treatment Technology Co., Wuhan, China) were set at each collection point. Every two traps are placed 500 m apart and run from 16:00–9:00 h the following day. The insects were collected directly into 70% ethanol and identified by morphology under a stereomicroscope (Leica, S APO), according to the keys and descriptions previously by Liu et al. [38]. During the period from July to September 2023, the frequency of collection was twice per month, and the total collection was six times in 3 months. After the morphological and species identification of culicids, every 20–30 *Culicoides* of the same species were divided into a batch for RNA extraction. Each batch of culicoides samples was added with 200 μL RNAiso-Plus (Takara, Cat#: 9109) and homogenized by the TissueLyser II homogenizer (Qiagen). The total RNA of samples was extracted following the manufacturer's directions of RNAiso-Plus and tested in parallel with the established real-time RT-MIRA and the RT-qPCR for AHSV.

### 2.10. Statistical Analysis

The GraphPad Prism (Version 9.0) and SPSS (Version 26.0) were used to statistical analysis, including the probit regression analysis and *κ* statistics. The statistical analysis's confidence interval was 95% (*p*  < 0.05).

## 3. Results

### 3.1. Results of the Optimal Primers and Exo-Probe

**Figure 1:**
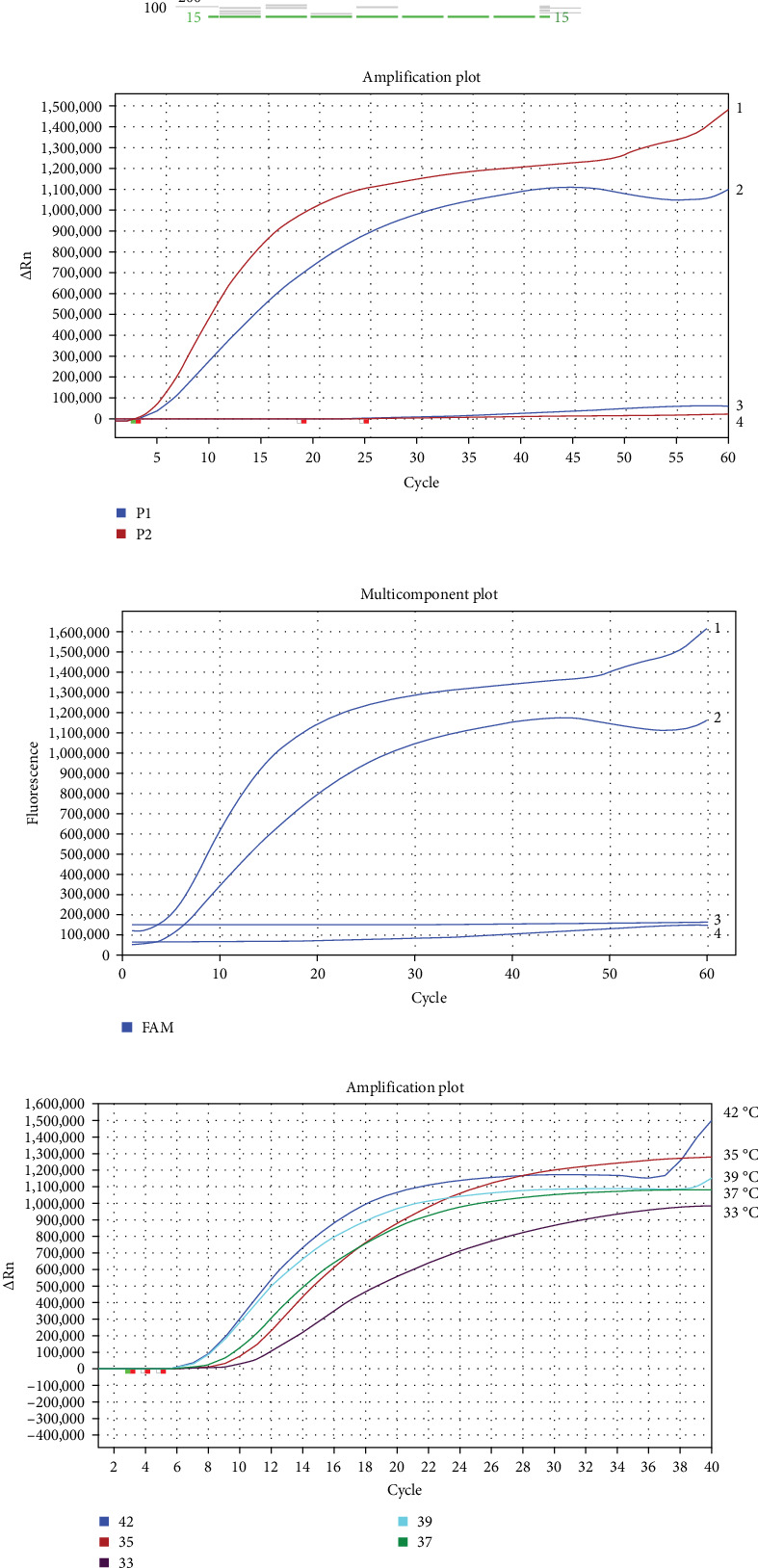
The screening of the amplification primers, exo-probes, and reaction temperatures for the ASHV real time reverse transcription multienzyme isothermal rapid amplification (RT-MIRA) assay. (A) The capillary electrophoresis diagram for the optimal RT-MIRA amplification primers screening. Lane 1 to 7, corresponding to the candidate primers African horse sickness virus (AHSV)-S7 1F/1R, 2F/2R, 3F/3R, 4F/4R, 5F/5R, negative control, and blank control. (B) The ampication plot diagram of two groups of primers and exo-probe. (C) The multicomponent plot diagram of two groups of primers and exo-probe. Curve 1 corresponds to the group of primers and exo-probe (4F/4R+exo-probe1), Curve 2 corresponds to the group of primers and exo-probe (5F/5R+exo-probe2), and Curve 3,4 correspond to the negative control of those two two groups of primers and exo-probe, respectively. (D) Optimization of real-time RT-MIRA assay at different temperatures. The curves of the real time RT-MIRA assay at temperatures, including 33, 35, 37, 39, and 42°C, respectively.

Five ASHV-specific primer pairs for RT-MIRA amplification were screened using pMD18-AHSV-vp7 as a template. The RT-MIRA amplified products were analyzed by a capillary electrophoresis device to determine the best specific primer pairs for RT-MIRA amplification for AHSV. According to the amplification results (Figure 1A), two pairs of specific primers (the pair 4F/4R and the pair 5F/5R) are the optimal primers and could be used in the next experiments.

Two exo-probes were designed based on the target regions (801–927 bp and 898–1123 bp) of the optimal primer pairs (4F/4R and 5F/5R). A real-time RT-MIRA kit was used to screen the optimal group of primers and exo-probe. These findings (Figure 1B,C) indicate that the group of the primer pair 4F/4R and exo-probe 1 had better performance, better amplification efficiency, and better fluorescence amplification curve compared with the group of the primer pairs 5F/5R and exo-probe 2. Therefore, the primer pair 4F/4R and exo-probe1 were the optimal prime and exo-probe combination of the real-time RT-MIRA for AHSV.

### 3.2. Optimization of Temperature for Real-Time RT-MIRA

For determining the optimal temperature, the real-time RT-MIRA assay's temperatures were, respectively, set at 33, 35, 37, 39, and 42°C, and the optimal primer-probe combination was used to detect the AHSV-positive RNA template with the real-time RT-MIRA kits. As shown in Figure 1D, although the amplification efficiency was higher at 42 and 39°C, their amplification curves were on an evident upward trend after the plateau. The fluorescence increment was the largest at 35°C, and its amplification curve was a typical S-shaped curve. Therefore, the optimal temperature for real-time RT-MIRA was at 35°C.

### 3.3. Workflow of the Real-Time RT-MIRA for AHSV

Through the above experiments, a rapid nucleic acid detection method for AHSV based on real-time RT-MIRA was established. Its workflow was as follows: First, a mixture of 29.4 μL Buffer A, 2 μL upstream primer (AHSV-S7-4 F, Table 1; final concentration 400 nM), 2 μL downstream primer (AHSV-S7-4R, Table 1; final concentration 400 nM), 0.6 μL exo-probe (Exo-probe1, Table 1; final concentration 120 nM), and 12.5 μL nuclear-free water was added into each reaction tube containing freeze-dried powder. Then, 1 μL of the nucleic acid samples were added into the reaction tubes, respectively, and the positive and negative controls were added in parallel. 2.5 μL Buffer B was added in the last step, and the reaction tubes were quickly turned upside down 5–6 times and rapidly centrifuged, then immediately incubated at 35°C for 20 min in a fluorescence detection device. The fluorescence signals were collected every 30 s with the FAM channel. In resource-limited diagnostic laboratories or outdoors, the amplification reaction could be performed in a portable warm bath device at 35°C or held by hand for 20 min, and the results were judged under a portable blue light device (480 nm).

### 3.4. Specificity of the Real-Time RT-MIRA for AHSV

**Figure 2:**
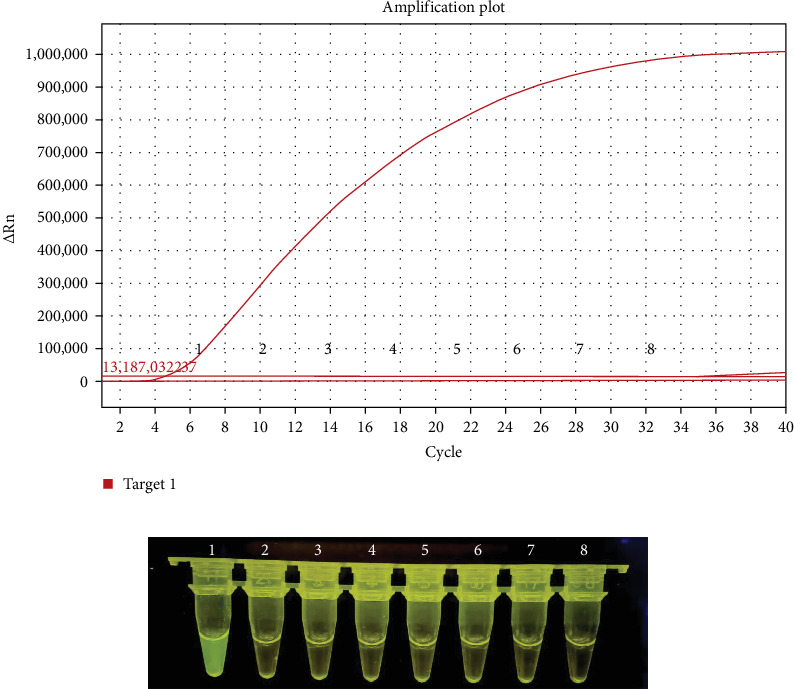
Sensitivity analysis of the real time reverse transcription multienzyme isothermal rapid amplification (RT-MIRA) assay for African horse sickness virus (AHSV). (A) The amplification results of real-time RT-MIRA in the ABI QuantStudio 5 qPCR System. (B) The visualization results of the real time RT-MIRA. Tubes or curves 1–8: RNA of AHSV, bluetongue virus (BTV), epizootic hemorrhagic disease virus (EHDV), pseudorabies virus (PRV), equine infectious anemia virus (EIAV), equine viral arthritis virus (EVAV), equine influenza virus (EIV), and ddH_2_O control, respectively.

For the specificity analysis, the RNA of other important viruses for equines or other Orbiviruse, including PRV, EIAV, EAV, EIV, BTV, and EHDV, were also tested by the real-time RT-MIRA method. The results showed that only AHSV RNA was positive and the other viruses were negative control were negative (Figure 2B). Besides, under the blue light (480 nm), only the amplification product of AHSV RNA were observed with strong green flourescence (Figure 2A). These results indicate that the developed real-time RT-MIRA method was specific for AHSV detection and had no cross-reactivity with other important equine viruses or other Orbivirus such as BTV and EHDV.

### 3.5. Sensitivity and Repeatability of the Real-Time RT-MIRA for AHSV

**Figure 3:**
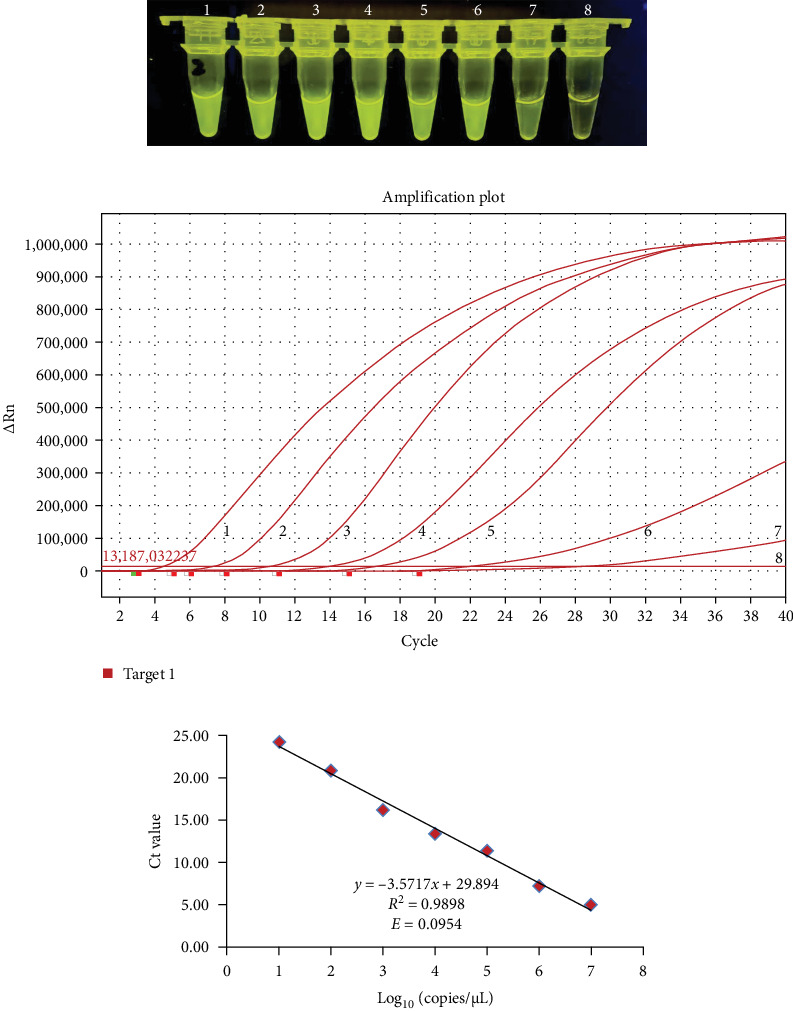
Sensitivity results of the real time reverse transcription multienzyme isothermal rapid amplification (RT-MIRA) assay for African horse sickness virus (AHSV). (A) Sensitivity results of the real time RT-MIRA assay by visualization observation. (B) Sensitivity results of the developed assay in a ABI QuantStudio 5 qPCR System. Tubes or curves 1–8: RNA templates corresponding to 1 × 10^7^ copies/μL to 1 × 10^0^ copies/μL, respectively. (C) The standard curves of the real-time RT-MIRA assay.

**Figure 4:**
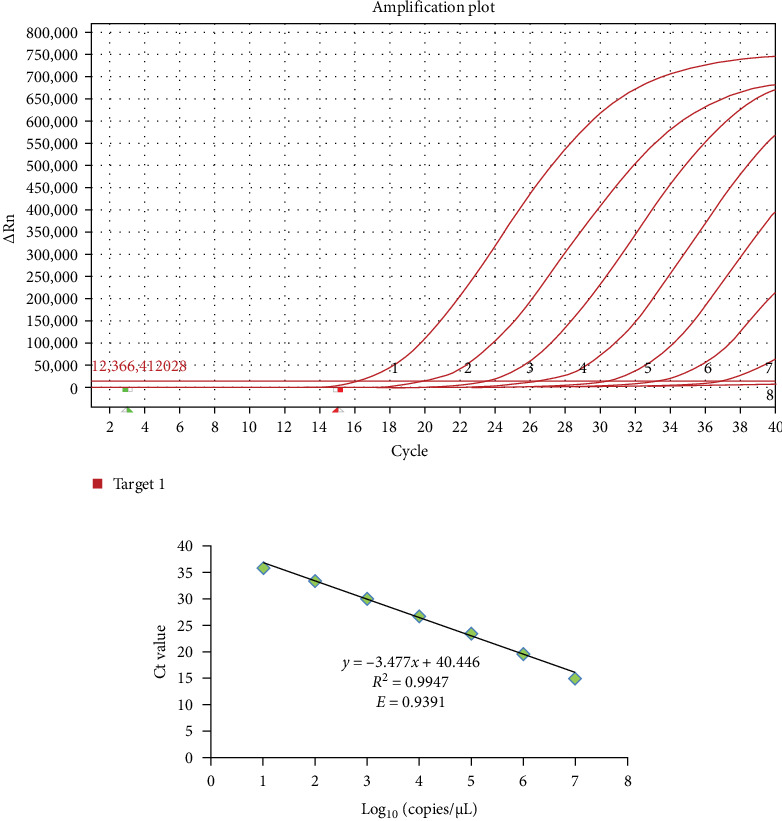
Sensitivity results of the RT-qPCR for African horse sickness virus (AHSV). (A) Sensitivity results of RT-qPCR assay commended by World Organization for Animal Health (WOAH). Curves 1–8: RNA templates corresponding to 1 × 10^7^ copies/μL to 1 × 10^0^ copies/μL, respectively. (B) The standard curves of RT-qPCR assay.

For evaluating the analytical sensitivity of the real-time RT-MIRA, the tenfold serially diluted AHSV RNA templates (1 × 10^7^ to 1 × 10^0^ copies/μL) were tested by the developed method and the WOAH recommended RT-qPCR for AHSV. Individual concentrations of the serial-diluted AHSV RNA were tested in quintuplicate. The sensitivity results of these two assays were shown in Figures 3 and 4. The results demonstrated that the limit of detection (LOD) of the real-time RT-MIRA assay was 10 copies/μL, which was consistent with that of RT-qPCR. Moreover, the real-time RT-MIRA amplification products of 1 × 10^7^ to 1 × 10^1^ copies/μL were observed obvious fluorescence at 480 nm blue light (Figure 3A). Furthermore, the real-time RT-MIRA for AHSV performed good repeatability as shown from the Ct values of five times repeated detection for individual diluted concentrations of the AHSV RNA template (Table 2).

In the real-time RT-MIRA, its standard curve had good linearity (*y* = −3.5717*x* + 29.894) with an *R*^2^ = 0.9898, the efficiency of the assay was 90.54% obtained by its slope (Figure 3C). The standard curve of RT-qPCR also had good linearity (*y* = −3.477*x* + 40.446) with an *R*^2^ = 0.9947, the efficiency of the RT-qPCR was 93.91% based on the slope of the standard curve (Figure 4B). Linear regression analysis indicated that the established real-time RT-MIRA could be applied for the quantitative detection of ASHV.

### 3.6. Compliance Testing for Simulated Samples

**Figure 5:**
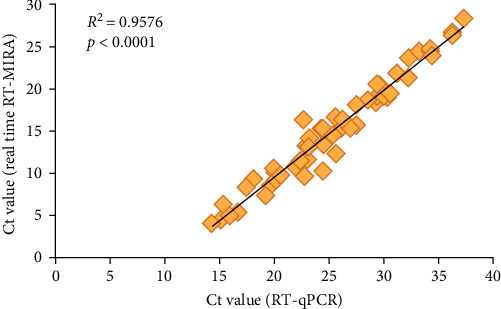
Correlation between the real-time reverse transcription multienzyme isothermal rapid amplification (RT-MIRA) and the RT-qPCR of African horse sickness virus (AHSV). There were 52 AHSV positive samples to be used to evaluated the correlation of these two methods. The cycle threshold (Ct) values of real-time RT-MIRA (*y* axis) and RT-qPCR (*x* axis) were analyzed by linear regression analysis, which results indicated there was a significant correlation between these two methods (*R*^2^ = 0.9576, *p*  < 0.0001).

For evaluating the performance of the developed real-time RT-MIRA for AHSV, 120 simulated samples were collected and simultaneously tested by the established method and RT-qPCR.  Table 3 showed the detection results of these two methods, which demonstrated that the overall agreement between the real-time RT-MIRA and RT-qPCR detecting the simulated samples was 98.33% (118/120). Compared with the RT-qPCR, the sensitivity, specificity, PPV, and FPV of the established method were 96.30% (52/[52 + 2]), 100% (66/[0 + 66]), 100% (52/[52 + 0]), and 97.06% (66/[2 + 66]), respectively. Furthermore, the statistical analyses indicated that there wes a good correlation between the two assays, with an *R*^2^ = 0.9576 (Figure 5) and a *κ* = 0.966 (*p*  < 0.001). The results of statistical analysis indicated that the established real-time RT-MIRA had a high level of consistency with the RT-qPCR. Beside, the overall agreement between the real-time RT-MIRA visual observation results and RT-qPCR detecting the simulated samples was 95.00% (114/120) and its *κ* value was 0.898 (*p*  < 0.001), indicating that the real-time RT-MIRA assay could be used for the visual detection for AHSV in outdoor.

### 3.7. Testing for Field Samples and *Culicoides* Samples

There were 396 field samples, including 256 horse blood samples and 140 donkey blood samples collected from Yunnan Province, Guangdong Province, and Hebei Province in China, which were tested in parallel with the established real-time RT-MIRA method and the RT-qPCR for AHSV. The results showed that all field samples were negative for AHSV (Table 4).

From June to September 2023, more than 1760 *culicoides* were collected from four sites (Guanming Farm, Safari Park, Xianhu Botanical Garden, and Mangrove Coastal Ecological Park) in Shenzhen of China, and the morphological identification showed that there were seven *Culicoides* species, including *Culicoides homotomus*, *Culicoides actoni*, *Culicoides arakawai*, *Culicoides circumbasalis*, *Culicoides oxystoma*, *Culicoides similis*, and *Culicoides peliliouensis*. However, 30 *Culicoides* were not identified due to incomplete morphology. Total RNA was extracted from 20 to 30 *Culicoides* of the same species and detected by the developed assay and RT-qPCR. As shown in Table 5, neither RT-MIRA nor RT-qPCR detected AHSV nucleic acid in these *Culicoides* (Table 5).

## 4. Discussion

AHS is a highly lethal viral infectious disease of equines. Once outbreak, it can cause a large number of naïve horses to die, thus, causing economic and animal welfare disasters. AHS is endemic to Southern African. However, due to a combination of globalization and climate change, the worldwide distribution of *Culicoide* species in the world has expanded rapidly, which may cause the spread of AHS in the world [39–41]. In February 2020, Thailand experienced the first AHS outbreak. Then in September of the same year, Malaysia reported an ASH outbreak to WOAH. The AHS outbreak in Thailand and Malaysia in 2020 was the second occurrence of the disease in Asia after a gap of 60 years, and also the first appearance of the disease in Southeast Asian countries. The outbreaks in these two countries indicate that AHS is breaking out of its original epidemic areas and spreading to Southeast and East Asia. Meanwhile, due to the humid and warm ecological environment in Southeast and East Asia, there are a large number of *Culicoide* species, which have a suitable ecological chain for AHS transmission. Furthermore, Southeast and East Asia have large numbers of horses naïved to AHSV. Taking China as an example, the number of horses, donkeys, and mules on hand in 2022 was 5.89 million, none of which have a history of AHS vaccination (https://data.stats.gov.cn/easyquery.htm?cn=C01&zb=A0D0N&sj=2022). According to statistics, there are 368 species of *Culicoides* in China [42], and 103 species of *Culicoides* are found in Yunnan Province adjacent to Thailand, making it the province with the highest number of *Culicoides* in China. As one important vector of AHSV, *C. Imicola* is found in Yunnan, Hainan, and Guangdong provinces of China [37, 43, 44]. In this regard, special attention should be paid to the prevalence of AHS in Thailand and Malaysia, and it is necessary to conduct surveys of AHS transmission vectors, monitoring the presence of AHSV in wild equine animals, and *Culicoides* in China's border areas. However, conventional detection techniques such as RT-PCR or real-time RT-PCR are not suitable for rapid screening of AHS in the field. Thus, it is urgent to establish a rapid, visualized, and reliable detection assay for AHSV.

As mentioned earlier, MIRA technology has many superiorities and is quite suitable for field surveys or rapid screening for human or animal epidemic diseases in resource-limited settings. Therefore, in this study, RT-MIRA-specific primers and exo-probe were designed, synthesized, and screened based on the AHSV *Seg-7* gene. The optimal specific primers and exo-probe obtained by screening were blasted with the *Seg-7* gene sequences of nine serotypes of AHSV, and the results showed that the region was highly conserved (Figure S1), which meant that the specific primers and probe combination were versatile and could be used to detect all nine serotypes of AHSV. In addition, the absence of AHSV in this study and the inability to reflect the RT process by using plasmid as the standard substance, we prepared a section of RNA of the AHSV *Seg-7* gene (641–1167 bp) by in T7 vitro transcription, evaluated the established real-time RT-MIRA using this section of RNA as a positive control. The valid test results showed that the established real-time RT-MIRA was highly specific for the detection of AHSV, without exhibiting any cross-reactivity towards other equine viruses or other *Orbivirus*; the LOD of the established assay was 10 copies/μL, which was consistent with that of RT-qPCR, meaning it had good sensitivity for detecting AHSV. Furthermore, the real-time RT-MIRA for AHSV performed good repeatability, and its standard curve exhibited good linearity with a correlation coefficient of *R*^2^ = 0.9898, which indicated that the established method could be used for the quantitative detection of ASHV. As no AHS infection cases have been reported in China, 120 simulated field samples were tested by the real-time RT-MIRA and RT-qPCR for AHSV, which results implied the diagnostic specificity and sensitivity of RT-MIRA were 100% and 96.30%, respectively. Furthermore, there was a significant correlation between the two methods, with a *κ* value of 0.966 and an *R*^2^ value of 0.9576. Additionally, among the 52 positive samples tested by the real-time RT-MIRA, 48 were judged as positive under a blue light instrument.

In the detection of field samples, this study tested 256 horse blood samples and 140 donkey blood samples from Guangdong, Hebei, and Yunnan provinces in China. The results of RT-qPCR and RT-MIRA showed that all samples were negative for AHSV. The survey results of *Culicoide* midges showed that there is *C. Imicola*, one of the important vectors for AHSV in Zhongshan City of China [44]. While Shenzhen City, adjacent to Zhongshan City, has the same climate and geographical conditions, and there are lots of horses and wild equine animals imported from overseas, it is necessary to investigate the species of *Culicoides* in Shenzhen and use the method established in this study to detect whether AHSV is carried in Culicoides. From July to September 2023, 1760 *Culicoides* were collected from four locations in Shenzhen (Wildlife Park, Xianhu Botanical Garden, Mangrove Coastal Park, and Guangming Farm). There were seven species of *Culicoides*, but no C. *Imicola* by morphological identification, which may be related to the investigation location and collection time. Neither RT-MIRA nor RT-qPCR detected AHSV in these *Culicoides*. The results of the *Culicoides* survey and detection for ASHV at four sites in Shenzhen for 3 months were not representative. However, in the detection process, the real-time RT-MIRA method showed advantages such as quick, convenience, and visualization, while its detection performance was consistent with RT-qPCR.

## 5. Conclusion

In this study, a real-time RT-MIRA assay based on ASHV Seg-7 gene was established and evaluated for AHSV detection. Compared with the WOAH recommended RT-qPCR for AHSV, the specificity and sensitivity of this method as good as the RT-qPCR. Moreover, this method has the advantages of fast and easy operation and is suitable for the rapid screening of AHSV. In addition, visualization of the results of real-time RT-MIRA assay makes it possible for an application to monitor the presence of AHSV in wild equine animals and *Culicoides* in border areas or grassroots farms.

## Figures and Tables

**Table 1:** The primers and probes used in African horse sickness virus (AHSV) real-time reverse transcription multienzyme isothermal rapid amplification (RT-MIRA) and RT-qPCR assays.

Name	Sequence (5′-3′)	Position	Source
ASHV-S7-1F	CGATAGCAGCAAGAGCCTTGTCCGTTGTAC	25–54	Designed in this study
ASHV-S7-1R	CCACAGTTGCCAACGCCTGATCATAATCTGGT	260–291	Designed in this study
ASHV-S7-2F	TCTGGCAGATATTACGTACCGCAAGGTCGAAC	429–460	Designed in this study
ASHV-S7-2R	GACGACATCTCCAGCCGCAACATTTACTCCAT	655–686	Designed in this study
ASHV-S7-3F	GTTGATGGAGTAAATGTTGCGGCTGGAGAT	651–680	Designed in this study
ASHV-S7-3R	GCCAAGTGGGAGAAACATACGCATAACATC	823–852	Designed in this study
ASHV-S7-4F	CACAAGATGTTATGCGTATGTTTCTCCCACT	818–848	Designed in this study
ASHV-S7-4R	CGCAACAATTTCATTCCTTTCAGTCGGTGG	915–944	Designed in this study
ASHV-S7-5F	CCACCGACTGAAAGGAATGAAATTGTTGCG	915–944	Designed in this study
ASHV-S7-5R	AGCATAAGAACCGACGCGACACTAATGAAA	1094–1123	Designed in this study
Exo-probe-1	GCACGCATTACGCGCTGTCATTTTTCAGCAGA (6-FAM-dT) (THF)AA (BHQ1-dT)ATGCAGCCTAT (C3 Spacer)	851–898	Designed in this study
Exo-probe-2	GTGTATGCGGCTTTGAGACCAGATTTCAGAA (6-FAM-dT) (THF)AA (BHQ1-dT)GGTGTTGTCGCGCCA (C3 Spacer)	972–1023	Designed in this study
RNA Template-F	TAA TAC GAC TCA CTA TAG GGT TTT GTC ACC GTT GAT GGA GTA	641–662	Designed in this study
RNA Template-R	GTA AGT GTA TTC GGT ATT GAC G	1146–1167	Designed in this study
AHSV-RT-qPCR-F	AGAGCTCTTGTGCTAGCAGCCT	1038–1059	[26]
AHSV-RT-qPCR-R	GAACCGACGCGACACTAATGA	1096–1116	[26]
AHSV-RT-qPCR-probe	6-FAM-TGCACGGTCACCGCT-BHQ-1	1080–1094	[26]

**Table 2:** Comparison of cycle threshold (Ct) values in African horse sickness virus (AHSV) RNA diluted for the same serial level in reverse transcription multienzyme isothermal rapid amplification (RT-MIRA) and RT-qPCR standard curves.

Initial concentration of quantified AHSV RNA	RT-MIRA					Ct mean ± SD	RT-qPCR					Ct mean ± SD
	Ct1	Ct2	Ct3	Ct4	Ct5		Ct1	Ct2	Ct3	Ct4	Ct5	
1 × 10^7^	5.18	5.24	5.73	5.92	5.74	5.56 ± 0.30	15.34	15.51	15.32	15.03	15.11	15.26 ± 0.17
1 × 10^6^	7.90	7.66	8.09	8.31	8.11	8.01 ± 0.22	19.89	19.88	19.52	19.92	19.84	19.81 ± 0.15
1 × 10^5^	12.80	12.97	12.02	12.66	12.76	12.64 ± 0.32	23.28	23.89	23.58	23.59	23.94	23.66 ± 0.24
1 × 10^4^	14.80	14.92	15.02	15.14	14.55	14.89 ± 0.20	26.62	26.68	27.08	27.02	26.97	26.87 ± 0.19
1 × 10^3^	18.17	17.92	17.62	18.34	17.77	17.96 ± 0.26	29.79	30.16	30.11	30.61	30.22	30.18 ± 0.26
1 × 10^2^	23.07	23.66	23.02	22.62	23.42	23.16 ± 0.36	33.20	34.08	33.93	33.15	34.23	33.72 ± 0.45
1 × 10^1^	26.32	25.51	25.34	29.13	28.84	27.03 ± 1.64	37.65	36.81	36.06	35.21	35.62	36.27 ± 0.87
1 × 10^0^	—	—	—	—	—	—	—	—	—	—	—	—

*Note:* “—” represents the unidentified value or invalid calculation result.

**Table 3:** Detection of African horse sickness virus (AHSV) in the simulated samples using the reverse transcription multienzyme isothermal rapid amplification (RT-MIRA) and RT-qPCR method.

Method		RT-qPCR			*κ*	*p*-Value
		Positive	Negative	Total		
Real-time RT-MIRA (real-time fluorescence readout)	Positive	52	0	52	0.966	<0.001
	Negative	2	66	68	—	—
	Total	54	66	120	—	—

Real-time RT-MIRA (blue light instrument)	Positive	48	0	48	0.898	<0.001
	Negative	6	66	72	—	—
	Total	54	66	120	—	—

**Table 4:** Results of field samples.

The sources of samples	Horse blood	Donkey blood
Hebei province	83	65
Yunnan province	121	32
Guangdong province	52	43
Results of real time RT-MIRA	Negative	Negative
Results of RT-qPCR	Negative	Negative

Abbreviation: RT-MIRA, reverse transcription multienzyme isothermal rapid amplification.

**Table 5:** Results of *Culicoides* samples.

Species	Total number	Collection site	Results of real time RT-MIRA	Results of RT-qPCR
*Culicoides homotomus*	126	1, 2, 3	Negative	Negative
*Culicoides actoni*	170	2, 3	Negative	Negative
*Culicoides arakawai*	482	1, 2, 3, 4	Negative	Negative
*Culicoides circumbasalis*	282	1, 2, 3, 4	Negative	Negative
*Culicoides oxystoma*	357	1, 2, 3	Negative	Negative
*Culicoides similis*	230	1, 2, 4	Negative	Negative
*Culicoides peliliouensis*	83	2, 4	Negative	Negative
Uncertain species	30	1, 2, 3, 4	Negative	Negative

*Note:* 1: Guanming Farm, 2: Safari Park, 3: Wutong Mountain Forest Park, 4: Mangrove Coastal Ecological Park.

Abbreviation: RT-MIRA, reverse transcription multienzyme isothermal rapid amplification.

## Data Availability

The data supporting the conclusions of this article are provided within the article. Raw data are available from the corresponding author upon request.

## Supporting Information

Supporting Information Figure S1 The blast results of ASHV-S7-4F, ASHV-S7-4R and Exo-probe-1 with AHSV Seg-7 of genotypes 1–9. The informations of AHSV genotypes are on the left. The positions of ASHV-S7-4F, ASHV-S7-4R and Exo-probe-1 are marked by black boxes, respectively. The positions of FAM and BHQ1 of the exo-probes are marked by solid black arrows, respectively.
