# Supplementary material for: Real-Time Reverse Transcription Multienzyme Isothermal Rapid Amplification for Rapid Detection of African Horse Sickness Virus
Source: Transbound Emerg Dis. 2025 Jan 13;2025:1852368. doi: 10.1155/tbed/1852368 (PMC12016855; doi:10.1155/tbed/1852368)
Supplement: Supporting Information — Figure S1 The blast results of ASHV-S7-4F, ASHV-S7-4R and Exo-probe-1 with AHSV Seg-7 of genotypes 1–9. The informations of AHSV genotypes are on the left. The positions of ASHV-S7-4F, ASHV-S7-4R and Exo-probe-1 are marked by black boxes, respectively. The positions of FAM and BHQ1 of the exo-probes are marked by solid black arrows, respectively. [file 1852368.f1.docx]

**Figure S1 The blast results of ASHV-S7-4F, ASHV-S7-4R and Exo-probe-1 with AHSV Seg-7 of genotypes 1–9.** The informations of AHSV genotypes are on the left. The positions of ASHV-S7-4F, ASHV-S7-4R and Exo-probe-1 are marked by black boxes, respectively. The positions of FAM and BHQ1 of the exo-probes are marked by solid black arrows, respectively.
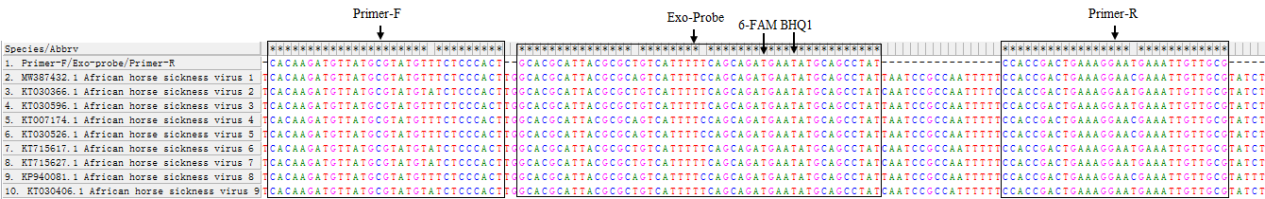


**Figure S1**
